# Quantitative Trait Loci Associated with the Immune Response to a Bovine Respiratory Syncytial Virus Vaccine

**DOI:** 10.1371/journal.pone.0033526

**Published:** 2012-03-15

**Authors:** Richard J. Leach, Ronan G. O'Neill, Julie L. Fitzpatrick, John L. Williams, Elizabeth J. Glass

**Affiliations:** 1 Department of Genetics and Genomics, The Roslin Institute and Royal (Dick) School of Veterinary Studies, The University of Edinburgh, Easter Bush, Midlothian, United Kingdom; 2 Department of Agriculture, Fisheries and Food, Celbridge, Co. Kildare, Ireland; 3 Moredun Research Institute, Edinburgh, United Kingdom,; University of Iowa, United States of America

## Abstract

Infectious disease is an important problem for animal breeders, farmers and governments worldwide. One approach to reducing disease is to breed for resistance. This linkage study used a Charolais-Holstein F2 cattle cross population (n = 501) which was genotyped for 165 microsatellite markers (covering all autosomes) to search for associations with phenotypes for Bovine Respiratory Syncytial Virus (BRSV) specific total-IgG, IgG1 and IgG2 concentrations at several time-points pre- and post-BRSV vaccination. Regions of the bovine genome which influenced the immune response induced by BRSV vaccination were identified, as well as regions associated with the clearance of maternally derived BRSV specific antibodies. Significant positive correlations were detected within traits across time, with negative correlations between the pre- and post-vaccination time points. The whole genome scan identified 27 Quantitative Trait Loci (QTL) on 13 autosomes. Many QTL were associated with the Thymus Helper 1 linked IgG2 response, especially at week 2 following vaccination. However the most significant QTL, which reached 5% genome-wide significance, was on BTA 17 for IgG1, also 2 weeks following vaccination. All animals had declining maternally derived BRSV specific antibodies prior to vaccination and the levels of BRSV specific antibody prior to vaccination were found to be under polygenic control with several QTL detected.

Heifers from the same population (n = 195) were subsequently immunised with a 40-mer Foot-and-Mouth Disease Virus peptide (FMDV) in a previous publication. Several of these QTL associated with the FMDV traits had overlapping peak positions with QTL in the current study, including the QTL on BTA23 which included the bovine Major Histocompatibility Complex (BoLA), and QTL on BTA9 and BTA24, suggesting that the genes underlying these QTL may control responses to multiple antigens. These results lay the groundwork for future investigations to identify the genes underlying the variation in clearance of maternal antibody and response to vaccination.

## Introduction

Infectious disease in livestock is a cause for great concern for both farmers and governments worldwide. Although many countries maintain good animal husbandry, farm management practices and vaccinate their livestock, failure in one or more of these control measures allows infectious disease to prevail [1]. More effective vaccines and the ability to breed for resistance have the potential to provide solutions for the control of both endemic and emerging or re-emerging infectious disease.

An understanding of the underlying genetics that control variation in immune responses and infectious disease outcomes may lead to the selection of more resistant animals, as well as identifying new strategies for improving vaccine efficacy. One example where genetic selection for improved resistance has the potential to make an impact is Bovine Respiratory Disease (BRD). Bovine respiratory disease has a complex aetiology caused by many different pathogens including viruses and bacteria [2]–[4] and affects cattle world-wide, resulting in major welfare problems and economic losses [5]. Both dairy and beef cattle show a wide range of clinical signs related to BRD, including nasal discharge, coughing, fever and decreased appetite when infected. There is evidence that the genetic makeup of the host contributes to the variation in BRD outcome although heritability estimates are low [5]–[9]. However, this evidence comes from field studies where the causal pathogen(s) were not identified, and thus the heritability of response to particular infections may be underestimated.

Bovine Respiratory Syncytial Virus (BRSV) is the most common viral pathogen implicated in outbreaks of BRD [10], [11], with an estimated 70% of calves in the UK becoming seropositive to the virus by 1 year of age [10]. Genetic factors have been shown to play a role in human susceptibility to the related pathogen, Human Respiratory Syncytial Virus (HRSV) [12], and as the epidemiology and pathology of HRSV and BRSV are similar [11], it is possible that at least a proportion of the genetic variation associated with BRD outcome [9], [13], may be related to the genetically controlled response to BRSV infection [14]. However, to date, no study of the genetic control of the response to a BRSV infection has been conducted in cattle.

Although vaccination is generally considered to be a useful means of controlling certain respiratory diseases in cattle populations, neither natural infection nor vaccination induces long lasting immunity [15] and young calves can be repeatedly infected. From studies of rodent models and humans, it has been suggested that a Th2 biased response may predispose the host to higher levels of pathology, whereas a T helper (Th) 1 biased response may be associated with protection [15]. Indeed, there are no licensed HRSV vaccines for humans, because following vaccination with a formalin-inactivated viral vaccine severe lung pathology was caused by natural infection [15]. Similarly in cattle there is evidence that an IgE Th2 biased response may be associated with greater clinical signs in both naturally infected calves and in response to formalin inactivated vaccines [16]. However, both modified live and inactivated viral vaccines are available for BRSV and are considered to be safe for use in cattle [14]. The efficacy of these vaccines is, however, low, especially in younger animals, with immature immune systems [17]. A further problem is that maternal antibody may also inhibit the induction of protection induced by vaccination [18].

Immunity to BRSV is generally considered to require neutralising antibody, but cellular immunity also plays an important role. However, cellular immunity may also induce pathology [19]. A fine balance between Th1 versus Th2 responses may be critical in determining the outcome of both BRSV vaccination and infection. In addition, an optimum level and timing of each type of response may be required to ensure that vaccines are protective and do not predispose to disease. The Th1 cytokine, interferon-γ (IFNγ) has been associated with protection against BRSV pathology [20]–[22] as has the corresponding Th1 antibody isotype, IgG2 [20]. The Th2 associated antibody isotype, IgG1, may be required for viral clearance and protection [23]. The production of IgE is generally considered to be part of a Th2 biased response, and has been associated with pathology in relation to BRSV infection and formalin inactivated viral vaccines [16]. However cytokines associated with both Th1 (IL-2 and IFNγ) and Th2 (IL-4) mediated responses were also up-regulated during BRSV infection [24]. It therefore seems likely that coordination of both Th1 and Th2 responses are required for optimal protection and reduced pathogenesis in BRSV infections in cattle.

In order to explore the factors underlying variation in the response of cattle to BRSV, a large study was conducted using young calves of a Charolais-Holstein cross population. A significant proportion of the variation of IgG1 and IgG2 responses to a live attenuated BRSV vaccine in these calves has been attributed to genetic factors as shown by sire and breed effects [25]. The clearance of maternal antibody to BRSV was also shown to have a genetic component. More recently we have shown that polymorphisms in one of the primary candidate loci implicated in the control of immune responsiveness, the Major Histocompatibility Complex (MHC) DRB3 gene, accounts for a proportion of the variation seen in response to the BRSV vaccine [26], however most of the genetic variation was shown to be controlled by non-MHC genes.

A long sought goal for animal breeders has been an ability to improve resistance to many pathogens. However it is not clear if this is a realizable goal. Identification of regions of the genome controlling responses to multiple pathogens or vaccine components may potentially make good targets for selective breeding and identify new pathways for improving vaccine efficacy in general. A previous immune-related Quantitative Trait Loci (QTL) study [27], used a foot-and-mouth disease virus (FMDV) peptide to elicit an immune response measured on the same animals as the current study using the BRSV vaccination. Thus we have also investigated if specific regions of the genome appear to control the response to BRSV vaccination in addition to the FMDV peptide.

In this paper we report a linkage analysis with 165 microsatellite markers to associate regions of the bovine genome with antibody isotype responses to a BRSV vaccine and compare the results with an earlier QTL study of response to a FMDV peptide carried out in the same population. QTL that account for a significant proportion of the variance in response to the BRSV vaccine were discovered, several of which overlap with those found in the FMDV study.

## Results

A total of 468 second generation (245 male and 223 female) animals of the RoBoGen Charolais×Holstein herd were phenotyped for total IgG, IgG1 and IgG2 responses to a BRSV vaccine over time. The whole herd (984 animals) was genotyped with 165 microsatellite markers.

Essentially all of the parameters of all the traits in this study have been previously described [25] (with exception of the addition of weight to the REML models, which was not found to be significant), but a summary table of the means, ranges and standard deviations of the traits are presented here for clarity (Table S1). Prior to vaccination, calves had declining levels of circulating BRSV-specific antibody, which was most likely maternally derived. After the initial vaccination, both IgG1 and IgG2 levels increased. The observed increase at each subsequent time point was significant (p<0.01) until a plateau was reached at day 35 for IgG1 and at day 49 for IgG2 (IgG1 = 38.96 ROD, IgG2 = 13.07 ROD. Table S1). Considerable variation in the levels of IgG1 and IgG2 was apparent between animals at all time points measured. However, the average IgG2 response remained lower than the average IgG1 response throughout the time course (Table S1).

### Correlations between anti BRSV: total IgG, IgG1 and IgG2

All traits were analysed for possible correlations with each other (pre vaccination correlations not shown) and many significant correlations within specific traits were found over time (Table S2). The IgG1 and IgG2 responses to BRSV vaccination at earlier time points correlated with their next corresponding time points. Thus the IgG1 level at week 2 correlated with the IgG1 level at week 5 (r^2^ = 0.26, p<0.01) and the IgG1 level at week 5 correlated with the IgG1 level at week 7 (r^2^ = 0.69, p<0.01). Similar correlations were found for the IgG2 levels. Furthermore, each time point correlated with the AUC for each respective isotype level, with the highest correlation found between the IgG2 week 5 post vaccination and the IgG2 AUC measurement (r^2^ = 0.79, p<0.01). The two BRSV specific IgG isotype levels were also significantly positively correlated with each other (Table S2). The highest correlation was between IgG1 and IgG2 at week 7 (r^2^ = 0.47, p<0.01). As expected, there were also negative correlations between the levels of antibody at week 0 and levels post-vaccination as the animals had pre-existing levels of IgG derived from colostrum, which inhibited the vaccine response [18], [25].

### QTL Results

**Figure 1:**
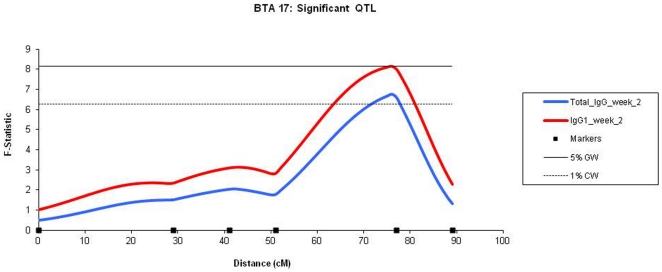
BTA 17: Significant QTL. *F*-statistic profiles for the total IgG and IgG1 response elicited by the BRSV vaccine two weeks post vaccination. The dashed horizontal line represents the threshold of the 1% chromosome wide significance level (*F* = 6.26) and the constant horizontal line represents the threshold of the 1% chromosomal wide significance level (*F* = 8.16).

A total of 27 QTL for BRSV response were discovered across 13 autosomes, with 9 QTL found on the initial genome scan and 4 QTL discovered when background effects were taken it account (Table 1). Seven QTL were above the 1% chromosome level, and of these, one QTL on BTA 17 was at the 5% genome wide significance level, explaining 3.47% of the phenotypic variance (σ_p_) (Fig. 1). The remaining 16 QTL were all above the 5% chromosome wide significance level.

**Table 1:** All QTL located in this study.

Chromosome^1^	Trait^2^	cM^3^	F^4^	a^5^	d^5^	Var%^6^	95% C.I. of QTLPosition^7^
2	Total_IgG_week_0	18 cM	5.47	−3.67**	6.22**	2.11	6–124 cM
2	IgG2_minus_week_2	19 cM	6.25	−4.22*	9.07**	2.67	60–124 cM
3	IgG2_minus_week_2	37 cM	5.19	1.89	6.77**	2.24	0–87 cM
3	IgG2_week_2	98 cM	4.88	1.03**	0.35	2.12	0–98 cM
7	IgG2_week_0	69 cM	5.21	0.74**	−0.1083	2.26	5–98 cM
7	Total_IgG_week_2	22 cM	4.92	3.97*	−4.8454	2.14	10–84 cM
8	IgG1_AUC	38 cM	5.9	26.36***	−3.34	2.52	6–118 cM
8	IgG1_week_7	44 cM	5.63	4.77***	0.32	2.42	0–118 cM
8	Total_IgG_AUC	43 cM	4.98	25.80**	−3.62	2.12	6–118 cM
8	Total_IgG_week_7	47 cM	5.53	5.63***	0.7997	2.40	0–118 cM
9	IgG2_week_2	45 cM	5.47	−0.87*	0.87	2.37	0–60 cM
10	IgG2_week_0	53 cM	7.01*	−0.07	−1.03***	3.01	48–138 cM
14	IgG2_AUC	15 cM	6.82*	13.67***	5.77	2.92	0–58.5 cM
14	IgG2_week_2	23 cM	5.79	0.99**	1.19*	2.51	6.5–60 cM
14	IgG2_week_7	16 cM	5.82	4.14***	−0.13	2.51	0–65.5 cM
15	Total_IgG_week_0	60 cM	4.85	3.64**	−2.73	2.10	0–80 cM
17	IgG2_minus_week_2	15 cM	4.88	−6.13	6.83	2.09	0–80 cM
17	IgG2_minus_week_2	57 cM	4.92	−6.44**	3.51	2.10	0–80 cM
17	IgG1_week_2	76 cM	8.17**	−4.30***	1.99	3.47	28–80 cM
17	Total_IgG_week_2	76 cM	6.67*	−4.20***	1.51	2.87	28–81 cM
18	IgG2_week_2	19 cM	7.29*	−3.58***	−2.20*	3.15	0–52 cM
18	IgG2_week_2	38 cM	5.81*	−0.96**	−0.90	2.52	18–52.5 cM
18	Total_IgG_week_7	18 cM	5.27	−4.67**	−1.83	2.28	0–67 cM
23	IgG2_AUC	27 cM	4.97	17.17**	1.71	2.15	0–80 cM
23	IgG2_week_2	31 cM	6.82*	2.03***	0.36	2.86	0–63 cM
24	IgG2_week_2	11 cM	4.15	0.16	1.48**	1.81	0–49 cM
28	IgG1_week_7	0 cM	4.37	3.41*	0.64	1.89	0–34 cM

1 Chromosome: the chromosome each QTL is located on.

2 Trait: each trait is shown as follows: total IgG or IgG isotype response, followed by the week relative to vaccination.

3 cM: the QTL position on the chromosome (centiMorgans).

4 F: the F-statistic of each QTL, all are at least 5% chromosome wide, * 1% Chromosome wide and ** 5% Genome wide.

5 “a” and “d” are the additive and dominance effects of each QTL, * = p<5%, ** = p<1% and *** = p<0.01%.

6 Var%: the additive phenotypic variance explained by the QTL.

7 The 95% confidence intervals of each QTL (centiMorgans).

Of the 27 QTL detected, 16 were associated with the IgG2 response (mean σ_p_ accounted for 2.5%), whilst 7 QTL were associated with the total IgG response (mean σ_p_ accounted for 2.3%) and only 4 with the IgG1 response (mean σ_p_ accounted for 2.6%). Thus loci accounting for a greater proportion of genetic effects controlling the phenotypic variance of the IgG2 antibody levels were identified in comparison to the total IgG and IgG1 antibody levels.

The traits in this study are linked (Total IgG is composed of IgG1 and IgG2), thus as several QTL co-located (the QTL peaks are within 20 cM of each other) it may be indicative that single QTL at these positions controlled several traits at different time points. If this is so, then there are 17 independent QTL (1 on BTA2; 2 on BTA3; 2 on BTA7; 1 on BTA8, BTA9, BTA10, BTA14 and BTA15; 2 on BTA17 and BTA18; 1 on BTA23, BTA24 and BTA28).

Nearly all of the QTL (23) identified showed significant additive effects (p<0.05), whereas only 7 showed significant dominant effects (p<0.05). Both Holstein and Charolais derived alleles were shown to have additive and/or dominance effects on QTL. Fourteen of the 27 QTL with significant additive effects had the Holstein derived alleles increasing the trait value (4 total IgG QTL, 2 IgG1 QTL and 8 IgG2 QTL); whilst 10 had Charolais derived alleles increasing the trait value (3 total IgG QTL, 1 IgG1 QTL and 6 IgG2 QTL) as shown in Table 1. Seven QTL had significant dominance effects, and of these 3 QTL had dominance effects with increases of the trait attributed to the Holstein alleles (all IgG2 QTL), whilst 2 QTL were traceable to Charolais alleles increasing the trait (Total IgG QTL and one IgG2 QTL). Overall, there did not appear to be a breed bias towards additive or dominance effects. However, the alleles derived from the Holstein breed appeared to increase the immune traits more frequently than the Charolais alleles in this study.

**Figure 2:**
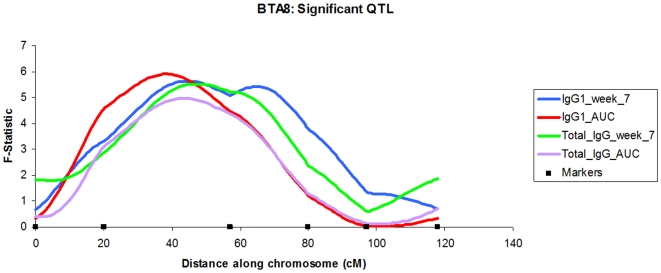
BTA 8: Significant QTL. *F*-statistic profiles for the total IgG and IgG1 responses elicited by the BRSV vaccine seven weeks post vaccination and using the AUC measurement. All the QTL are above the 5% chromosome wide significance level.

The QTL associated with total IgG levels tended to overlap (+/− 7 cM of the peaks) with either IgG1 or IgG2 QTL. Five of the seven total IgG QTL overlapped (BTA2, two on BTA8, BTA17 and BTA18; Table 1), reflecting the fact that the total IgG phenotype was made up from the components of the IgG1 and IgG2 phenotypes. These overlapping QTL also included an overlap between QTL associated with total IgG AUC and the IgG1 AUC on BTA 8 (Fig. 2).

A total of 8 QTL were associated with the IgG levels prior to vaccination, whereas the majority of QTL were associated with the response post vaccination and were mostly on different chromosomes from those associated with the pre-vaccination levels (Table 1). The trait with the greatest number of different QTL associated with it was the IgG2 response 2 weeks post vaccination, with a total of 7 QTL, in contrast to the single QTL associated with the IgG1 response at the same time point. Fewer QTL were associated with the antibody response following the vaccine boost, with only one associated with the IgG2 response at week 7, but two for the IgG1 response at this time point.

**Figure 3:**
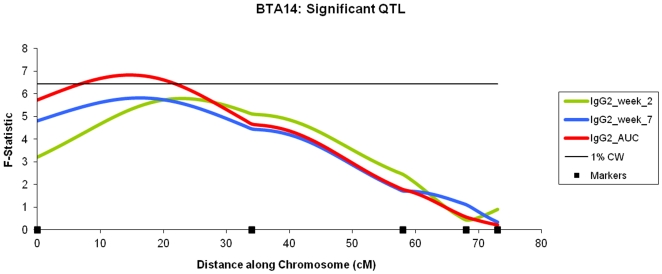
BTA 14: Significant QTL. *F*-statistic profiles for the IgG2 response elicited by the BRSV vaccine two and seven weeks post vaccination and using the AUC measurement. The constant horizontal line represents the threshold of the 1% chromosomal wide significance level (*F* = 6.43).

**Figure 4:**
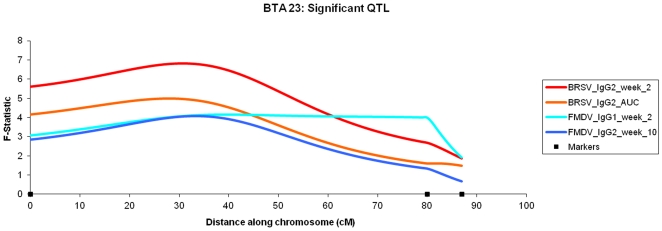
BTA 23: Significant QTL. *F*-statistic profiles for the IgG2 response elicited by the BRSV vaccine two weeks post vaccination (peak, 31; F-statistic 6.82) and the AUC measurement (peak, 27; F-statistic, 4.97) and for the IgG1 (peak, 39; F-statistic, 4.16) and IgG2 (peak, 33; F-statistic, 4.08) levels elicited by the FMDV peptide [27] 2 and 10 weeks post immunisation, respectively.

The most significant QTL were mainly associated with the IgG2 responses, with 5 of the 7 QTL above the 1% chromosome significance level (including the QTL also at the 5% genome wide significance level). These QTL also had other QTL within the confidence intervals for differing traits and time points (with the exception of the QTL located on BTA 10, Table 1). For example: BTA 14 contains a QTL associated with the IgG2 AUC measurement at the 1% chromosome wide significance level (Fig. 3) and within 8 cM of the peak of this QTL are QTL for IgG2 week 2 and IgG2 week 7 post vaccination. In addition, another 2 QTL associated with the immune response post vaccination (IgG2 week 2, 1% chromosome wide and the IgG2 AUC measurement, 5% chromosome wide) were discovered on BTA 23, with their peaks separated by 5 cM (Fig. 4).

Fourteen of the QTL identified in this study were within the confidence intervals of QTL detected in the FMDV study [27]. Furthermore, the peaks of four of these QTL were within 10 cM of QTL peaks associated with the response to the FMDV peptide and included BTA 9 at 45 cM, BTA23 at 27 cM and 31 cM and BTA24 at11 cM. Of the overlapping QTL, the QTL on BTA 23 were the most striking, as two QTL from both studies overlapped across the same regions of BTA23 (Fig. 4) which contains the MHC region.

## Discussion

In this study we have discovered a number of distinct QTL which contribute to the control of the immune response to BRSV vaccination as well as clearance of maternal antibody in cattle. Although there was evidence for QTL within the MHC region on BTA23, the majority of the QTL described in this paper do not lie within this region. Our evidence suggests that some chromosomal regions may control both the IgG1 and IgG2 response. However, most of the QTL detected appear to play a role in the IgG2 response. This study is a follow on from our previous study of the same crossbred herd which found strong heritability (h^2^ = 0.52 with respect to total IgG levels over time (day0–day35)) and significant sire effects in the IgG response post vaccination [25]. In the present study 27 significant QTL were associated with BRSV specific IgG1, IgG2 and total IgG levels, pre- and post-vaccination, which were located on 13 bovine autosomes. The multigenic nature of the response must at least in part, reflect the complexity of host immunity.

In the previous study the Holstein backcross calves were shown to exhibit significantly higher antibody levels than the Charolais backcross calves pre-vaccination but not post-vaccination [25]. The QTL found in the current study did not reflect this, as both breeds had similar QTL additive effects, although the Holstein backcrosses generally had higher antibody levels pre-vaccination. This finding is in contrast to a study which investigated bovine keratoconjunctivitis [28], where the additive effects of QTL originated from a breed with known low prevalence of the disease [29]. This suggests that further QTL are yet to be discovered, which may explain the higher antibody levels in the Holstein backcross cattle pre-vaccination. Furthermore, most of the significant dominance effects of QTL associated with pre-vaccination levels, were derived from the Charolais breed, suggesting a complex mode of inheritance may be responsible for the observed phenotypes, such as parent-of-origin effects [30] or over-dominance [31]. Further studies would be warranted to investigate these dominance effects as breeding programs only consider additive effects [32].

The persistence of circulating maternal antibodies has been shown to have an effect on the efficacy of vaccination against several important infectious diseases [33]. It is considered preferable to vaccinate calves against BRSV at an early age, because it is prevalent and can cause serious clinical disease, even though maternal antibodies affect BRSV vaccine efficacy [34]. In the present study the levels of anti-BRSV IgG were declining prior to vaccination, a pattern most likely due to the clearance in the levels of circulating maternal antibody [18], [25]. Thus the process of detecting QTL for antibody response post-vaccination may have also been affected by the unknown effect of maternal antibody on the efficiency of immunisation.

Eight QTL were detected associated with the BRSV-specific antibody levels pre-vaccination. Two of these on chromosomes 10 and 15, were not associated with the response post-vaccination, while the others were on chromosomes with post-vaccination QTL, but with apparently different peaks. These QTL may therefore reflect genes/pathways which impact on the persistence of maternal antibody. The transfer of IgG from dam to calf via colostrum has high heritability [35]. The results presented here suggest that IgG transfer may be partly controlled by the genetics of the calf. Furthermore, the neonatal Fc receptor, a heterodimer consisting of a MHC class I α-chain homolog located on BTA18 [36] and beta-2-microglobulin (b2m), located on chromosome 10 [37], may play a significant role in regulating the level of maternal antibody [36]. Polymorphisms in the MHC α-chain homolog on BTA 18 have been associated with the levels of IgG in newborn calves [36]. However the QTL detected on BTA18 were associated with the post-vaccination response and not pre-vaccination levels. Furthermore, the gene for the MHC α-chain homolog is located outside of the final marker on that chromosome, and thus outside the confidence intervals for these QTL; further genotyping in this region may be able to provide better resolution. In addition, the gene encoding b2m, which has been associated with the failure of transfer of maternal IgG to calves [40] is positioned just outside of the confidence interval of the pre-vaccination QTL located on BTA10 which was the only QTL detected in the current study on this chromosome. Addition of markers to the analysis of BTA 10 may better define the QTL position and determine whether b2m is a putative candidate gene controlling pre-immune IgG. Identifying the genes underlying the QTL associated with variation in levels of maternal antibody could potentially aid the design of future vaccines to minimise the interference effects of maternal antibodies.

A single QTL was identified, located on BTA 14, which influenced the response at different time points, which appears to influence the IgG2 response at 2 and 7 weeks post vaccination as well as the overall (AUC) IgG2 response post vaccination. This is in marked contrast to the 8 clusters of QTL that were associated with the response to the FMDV peptide over time [27]. However more time points were measured in the FMDV study which may have increased the chances of detecting QTL over time. Also the FMDV antibody levels were measured with higher resolution and were not influenced by maternal antibody, both of which may have reduced the ability to detect BRSV related QTL in the current study. No T cell response was detected post vaccination (results not shown). Although it is clear that cell mediated immunity is important for protection against BRSV [15], generally peripheral blood BRSV-specific T cell responses to modified-live vaccines are only detected in seronegative calves [38] or in experimental vaccines formulated to potentiate cellular immunity (e.g.[39] and [21]). Nonetheless the data indicate that distinct genes operate early in the primary BRSV vaccine response compared to those that underlie variation following the boost. The QTL associated with the IgG2 response at week 2 following the primary vaccination also accounted for the most phenotypic variance. In addition, the IgG2 response at week 2 had the most QTL associated to it within this study, which altogether explained over 17% of the IgG2 response at this time point. As the responses at later time points also correlate with the week 2 response, this suggests that these QTL may be particularly important in determining the overall IgG2 response. In contrast, no QTL were identified which were associated with either IgG isotype, 5 weeks post vaccination. Previously, it has been shown that the variation in IgG1 and IgG2 at this time point is small among the different second generation classes (F2 CB1 and HB1) [18]. Understanding the genetic control of the variation in the IgG2 response (associated with Th1 responses) at early time points, may suggest new ways to modulate the Th1 and Th2 balance induced by vaccination, which may be relevant for improved protection and avoidance of adverse pathology following vaccination [15], [16]. Markers associated with these genes would be valuable for breeding for enhanced disease resistance.

In contrast to the response to the FMDV peptide [27], the anti BRSV IgG1 and IgG2 levels were highly correlated, both pre and post vaccination, however, no QTL for IgG1 levels were identified that overlapped with QTL for IgG2 levels, which is in contrast to the QTL for FMDV peptide response where three chromosomal regions on BTA 20, 23 and 25 had QTL peaks for both anti-FMDV peptide IgG1 and IgG2 responses [27]. These findings imply strong correlation seen at the phenotypic level does not necessarily indicate that the phenotypes share genetic control. Thus although the levels of both antibody isotypes for BRSV increased at similar rates post-vaccination, different genes may be involved in controlling the response of IgG1 and IgG2. Although cellular and humoral immunity play important roles in response to BRSV, it remains unclear how the contribution of different isotype subclasses of bovine immunoglobulin and components of cell mediated immunity, such as cytokines, impacts protection and pathology in relation to BRSV [15]. Available evidence suggests that a relative balance between Th1 and Th2 responses determine the outcome of infection and vaccination, and that pathology can be associated with an exacerbated Th2 response involving IgE [16]. Our study design did not allow us to investigate protection and pathology, and further studies are warranted to explore their relationship with genetics of immune response. Nonetheless our results suggest that host genetics may play a role in determining the balance between Th1 and Th2 responses as well as explaining a proportion of the considerable between animal variation. Understanding the genes underlying regulation of Th1 and Th2 pathways could suggest new ways to immuno-modulate responses to pathogens such as BRSV. Furthermore, the markers identified here could be useful in marker assisted selection to select, for example, animals with a lower Th2 response, thus potentially reducing pathology when infected with BRSV.

Almost half of the 27 QTL significantly associated with IgG levels pre and post BRSV vaccination, lie within the 95% confidence intervals of the QTL associated with antibody responses to the FMDV peptide [27]. Indeed, 10 of these QTL are associated with the same IgG isotype, suggesting that the same genes influence IgG levels post immunisation with different immunogens. Furthermore, QTL identified in the current study also fall within the confidence intervals of QTL associated with other immune traits, including: the change of somatic cell score in response to Mastitis [41] on BTA15; antibody response to *Mycobacterium avium ssp. paratuberculosis* [42] on BTA 8 and 9; two thirds of the haplotypes significantly associated with Bovine Viral Diarrhea and eight SNP associated with BRD [43] on BTA2; and bovine spongiform encephalopathy infection [44] on BTA 17. This raises the possibility that the immune responses to different stimuli may be controlled, to some degree, by similar pathways, even if the initial detection of the infection occurs through distinct mechanisms, downstream signalling may converge on similar pathways. If the genes underlying these QTL are involved in pathways that control responses to a wide range of pathogens they may be suitable for selective breeding for disease resistance in general. In addition, understanding of the underlying genes and pathways could point to new targets for future immunomodulatory substances such as adjuvants.

The MHC locus is expected to be a significant component underlying variation in immune response [45], as it contains many polymorphic and immune-related loci [46], [47]. Indeed in both the current study and the FMDV peptide study [27] QTL were discovered that span the MHC locus. Indeed, earlier studies have shown that polymorphisms at the *DRB3* locus are associated with response to BRSV [8], [48] as well as protection against viral challenge following immunisation with the FMDV peptide and similar peptides [49]. However, the two QTL that span the MHC loci that were identified in the present study only account for 3% of the phenotypic variation, which together with previous data from the FMDV study [27], indicates that many other regions of the genome are involved in controlling variation in immune responses.

The human strain of respiratory syncytial virus (HRSV), which is closely related to BRSV, is a major cause of respiratory disease in young children, where the epidemiology and pathogenesis of infection is similar to that of BRSV [10]. The severity of disease in young children caused by HRSV is significantly associated with genes expressed during both innate and acquired immune responses [50]. Eight of the twenty one genes implicated in the human response to HRSV [50], have orthologs within the QTL in the current study, on BTA 2, 3, 15, 17 and 23. These are STAT1 (BTA 2), TNF (BTA 23), VCAM1 (BTA 3) and IL15 (BTA17) which play roles in innate immunity and CD28 (BTA 2), STAT1, IL17 (BTA23) which play roles in adaptive immunity [51]. The QTL with the highest significance in the current study (on BTA 17) encompassed IL17 within its 95% confidence intervals. Two further genes, from the *Janssen et al* study [50], within these QTL confidence intervals (MS4A2; FCER1A, both located on BTA 15) are associated with asthma allergies as well as severe RSV disease. It has also been suggested that a Th2 biased response in cattle, associated with induction of IgE, is correlated with pulmonary pathology in cattle [16], and thus polymorphisms in these genes may also influence the pathology observed in young cattle infected with BRSV. Further study on the role of genetic control of IgE responses may also highlight these genes and others associated with pathology. It is interesting to note that some of the SNPs within IL15, STAT1, VCAM1 and TNF associated with HRSV in the human study [50] are also polymorphic in the bovine genome, making them very good candidates for further study

This is the first time that the natural variation in the clearance of maternal antibody and the immune response to a BRSV vaccine has been successfully exploited to locate significant QTL in distinct regions of the bovine genome. A greater number of QTL were found associated with IgG2 response and these explained a larger part of the genetic variation than were found for IgG1 response. Some of the QTL identified overlap with QTL associated with the response to a FMDV peptide. Research is ongoing to further reduce the QTL confidence intervals, with the aim of identifying the underlying gene variants. This information may be used to breed for disease resistance and the production of more efficacious vaccines through the discovery of key genes and gene pathways.

## Materials and Methods

### Ethics Statement

All animals were clinically normal and all experimental protocols were authorised under the UK Animals (scientific procedures) Act, 1986. In addition, The Roslin Institute's Animal Welfare and Ethics Committee (AWEC) ensure compliance with all relevant legislation and promote the adoption and developments of the 3Rs (reduction, replacement, refinement).

### Animals

A total of 501 cross-bred second generation animals were produced in the RoBoGen herd. Pure-bred Charolais sires were mated to Holstein dams to produce the F1 (137 animals) and 8 F1 sires were mated to F1 heifers to create the F2 (315 animals) of the second generation. In addition the founder Charolais sires were mated to F1 heifers to produce a Charolais backcross (CB1, 88 animals) and F1 sires were mated to pure bred Holstein heifers to create a Holstein backcross (HB1, 98 animals). Thus the second generation consisted of the F2, CB1 and HB1. The whole pedigree consisted of 21 sires (8 F0, 13 F1) and 408 dams (131 F0, 277 F2). All male calves had unrestricted suckling with their dams, at grass, until approximately 4 months old. All female calves were weaned by 36 h, segregated from the rest of the herd and raised indoors, initially on milk-replacer then weaned early onto a propriety compound diet. Any differences in the results observed between sexes were therefore confounded by differences in management.

### Vaccination with Rispoval and sampling

Immune response measurements were collected on 468 second generation crossbred calves (294 F2, 90 HB1 and 81 CB1). The age at first immunisation with the BRSV vaccine (Rispoval ©, Pfizer Animal Health, Surrey, UK) ranged from 88–195 days and fell within the recommended vaccination period according to the datasheet provided by the manufacturer. All animals were vaccinated and IgG levels measured as described by O'Neill *et al* [25]. Each animal received 2 ml of the attenuated live vaccine (the vaccine contained no adjuvant), intramuscularly, according to the manufacturer's recommendations. All calves were re-immunized with 2 ml of the vaccine intramuscularly, 3 weeks following the initial vaccination. Blood samples were collected by jugular venipuncture: 4 and 2 weeks pre-vaccination; at week 0 (the day of first vaccination) and at 2, 5 and 7 weeks post-vaccination, providing six samples per calf. Serum was collected within 2 h of sampling and stored at −20°C until testing.

### ELISA for detection of BRSV-specific total IgG, IgG1 and IgG2

This assay was carried out as described in O'Neill et al, 2006 [25]. Briefly, sera were tested quantitatively by solid-phase antibody capture ELISA specific for total BRSV-IgG according to the manufacturer's guidelines (Svanovir BRSV-Ab, Svanova Biotech, Uppsala, Sweden). Samples were tested in duplicate, at a dilution of 1/25 and optical densities (OD) read at 450 nm. The corrected optical density value for each sample and the control sera was calculated by subtraction of the OD value of each control antigen-coated well from that of the corresponding viral antigen-coated well and the relative optical density (ROD) value for each sample was calculated as a proportion of a positive control serum on a per-plate basis.

BRSV-IgG2 levels were tested using a modified form of the above assay. All dilutions were as described above but 100 µl of 1/10,000 horse-radish peroxidase-conjugated anti-bovine IgG2 (Acris Antibodies GmBH, Hiddenhausen, Germany) was used as the secondary antibody. The corresponding ROD values were also calculated as above. To obtain the IgG1 levels, IgG2 was subtracted from the total IgG, as described in O'Neill et al, 2006 [25].

Area under the curve (AUC) for each trait was also calculated (using the trapezoidal rule [52]) to provide a single trait that reflected the overall response of each animal.

### Immunisation with the FMDV15 peptide

Full details of the FMDV peptide and the sampling protocols have been previously published [27]. The peptide consisted of two separate regions (residues 141 to 158 and 200 to 213) of the virus coat protein (VP1) from the O1 Kaufbeuren strain of FMDV [53]. 195 second generation cross heifers (121 F2, 43 HB1 and 31 CB1) were immunised subcutaneously with 1 mg FMDV15 peptide/animal emulsified in Freund's incomplete adjuvant at week 0, followed by a boost of 100 µg FMDV15 peptide/animal at week 6. The age at initial immunisation with the peptide ranged from 469–609 days. Thus the FMDV immunisation and collection of phenotypes were done subsequently to the BRSV vaccination and collection of phenotypes. Whole blood samples were collected by jugular venipuncture from all of the female F2 and backcross heifers, post immunisation at weeks 0, 1, 2, 4, 8 and 10 for IgG1 and IgG2 analysis. Animals used in the FMDV study were the same female animals used in the BRSV study reported here.

For the IgG analysis, the blood samples were allowed to clot and serum collected and stored at −20°C until they were tested. FMDV15 peptide specific ELISAs were performed to measure IgG1 and IgG2 isotypes as detailed in Baxter *et al* 2009 [25]. ELISAs were conducted within a short period following the final sampling, to minimise technical variation.

### Statistical Analysis – BRSV IgG

The IgG concentrations were log_10_ transformed to obtain a normal distribution and constant variance, to permit REML (REsidual Maximum Likelihood) analysis.

Significant factors, such as sire, within this study have been previously calculated by O'Neill *et al* [25]. For the QTL calculation, the final model included sire and dam as random effects; the fixed effects within the model, with appropriate degrees of freedom (df), were *line* (F2, CB1, HB1; 2d.f), *age* (age at vaccination), *cohort* (1, 2, 3; 2d.f) and *calculated weight* (weight of the animal at first vaccination date calculated from regression of animal weight at other time points pre and post vaccination).

Thus the model was:
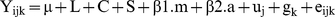
Where: Y_ijk_ is the observed value of the phenotypic trait; μ, population mean; L, the fixed effect of line (F2, CB1 or HB1); C, the fixed effect of cohort (3 Cohorts); S, the fixed effect of sex (male or female); β1, the linear regression on the covariate of age at vaccination, m (m = d469–d609); β2, the linear regression on the covariate of weight, a (a = 361–744 kg); u_j_, the random effect of sire; g_k_, the random effect of dam g; e_ijk_, the residual error e∼N(0,Iσ^2^_p_).

### Correlations

The residuals, for each time point, from the Restricted Maximum Likelihood (REML) models were saved and used to calculate all the correlations between the immune responses at differing time points throughout the study.

### Genetic Markers

Standard phenol-chloroform methods were used to extract DNA from blood samples [54]. A panel of microsatellite markers were genotyped across all individuals in the herd, with a total of 165 microsatellite markers distributed across all 29 autosomes [55]. All of the genotypes were stored in the ResSpecies database [56] and used to build linkage maps with CRIMAP 2.4 [57]. The maps were compared to a bovine linkage map [58]. Once inconsistencies with the published linkage maps were resolved, the maps constructed using CRIMAP were used to conduct putative QTL analysis for the immune-related traits. The map is given in Table S3.

### QTL Analysis

GridQTL [59] an internet based software was used for the QTL analysis, the F2 and backcross module was used which assumes that founder lines are fixed for alternative alleles at QTL loci (although they can be segregating at markers) and implements a least squares analysis. Information content (IC) along the linkage maps was also calculated by the program. Both single and two QTL models with additive and dominance effects were fitted at 1 cM intervals along the autosomes, using a sex averaged genetic map. By setting the two founder breeds as: breed 1 = Holstein and breed 2 = Charolais, the positive or negative sign of the effects (additive and dominance) dictated the origin of the allele (Holstein or Charolais) which increased the trait value. Thus, when the additive effect was positive, Holstein origins were responsible, and when the additive effect was negative then Charolais origins were responsible. However, when dominance effects shared the same sign as the additive effects, Holstein origins were responsible, and when the signs did not match, Charolais origins were responsible for the increased phenotype.

In total 21 phenotype measurements (for total IgG, IgG1, IgG2 at weeks 4 and 2 pre-vaccination and 0, 2, 5, 7 post vaccination along with AUC measurements) were tested for linkage association using 165 microsatellite markers spread across all 29 autosomes in the bovine genome. Significance thresholds were calculated by permutation analysis with 2000 permutations [60]. Four significance levels were used: chromosome-wide 5% and 1% and genome-wide 5% and 1%.

### Refining QTL

The QTL detected at the 5% chromosome-wide significance level and above were included in the model and the genome rescanned for further QTL. By adding the initial QTL as background effects, the variance caused by them is removed, thus potentially revealing previously undetectable QTL. In cases where more than one QTL was found for the same trait on the same chromosome, a 2 QTL model was applied by fitting two QTL simultaneously and re-analysing the data. A forward and backward selection interval mapping approach was used to check whether QTL moved significantly [61]. These refining methods were repeated, until no further QTL were detected. Finally, bootstrap analysis was performed, using 2000 repeat samples, for all chromosomes, where significant QTL were detected, to estimate the 95% confidence intervals for the location of the QTL [62].

## Supporting Information

Table S1 **Phenotype summaries.** 1. Immunisation with FMDV peptide (data from previous study [27]); BRSV vaccine (data from previous study [25]). 2. Trait: each trait is shown as follows: total IgG or IgG isotype response, followed by the week relative to vaccination. 3. Mean: mean average of each time point. BRSV specific antibody is measured as Relative Optical Density whilst FMDV specific antibody is measured in µl/ml. 4. Range: Minimum and maximum response for each time point (units as 3.). 5. SD: the standard deviation of each trait mean at each time point.
(TIF)

Table S2 **Correlations within and between the immune responses to the BRSV vaccine.** The x and y axes consist of each week of the BRSV study [27]. Correlations are located below the black shaded boxes. Above the black shaded boxes is the significance of each correlation. The horizontal orange (IgG1 AUC measurement), green (IgG2 levels two post vaccination), red (IgG2 levels 5 weeks post vaccination), blue (IgG2 levels 7 weeks post vaccination) and purple (IgG2 AUC measurement) shaded boxes represent the significant correlations throughout the BRSV study. The vertical coloured shaded boxes highlight the corresponding significance of the correlations.
(TIF)

Table S3 **Linkage map.** Marker distances (cM Kosambi) are shown for the sex-average maps built for the Charolais×Holstein population used in this study.
(TIF)
