# Supplementary figures and images for: Quantitative Trait Loci Associated with the Immune Response to a Bovine Respiratory Syncytial Virus Vaccine
Source: PLoS One. 2012 Mar 15;7(3):e33526. doi: 10.1371/journal.pone.0033526 (PMC3305305; doi:10.1371/journal.pone.0033526)

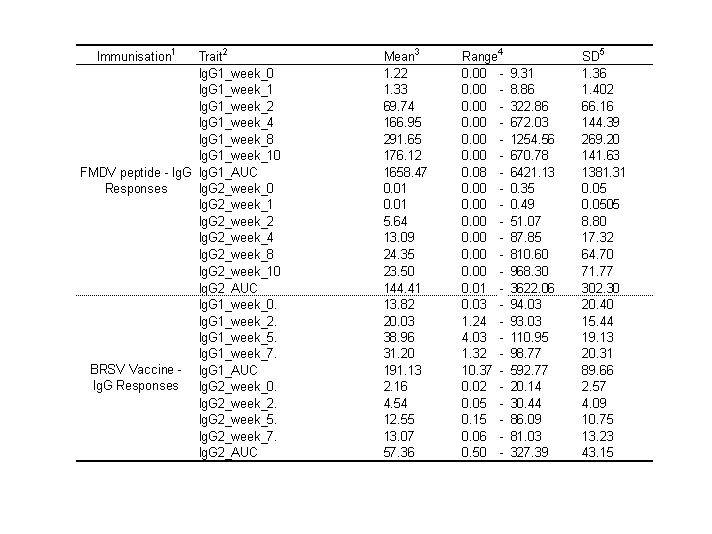

Supplement: Table S1 — Phenotype summaries. 1. Immunisation with FMDV peptide (data from previous study [27]); BRSV vaccine (data from previous study [25]). 2. Trait: each trait is shown as follows: total IgG or IgG isotype response, followed by the week relative to vaccination. 3. Mean: mean average of each time point. BRSV specific antibody is measured as Relative Optical Density whilst FMDV specific antibody is measured in µl/ml. 4. Range: Minimum and maximum response for each time point (units as 3.). 5. SD: the standard deviation of each trait mean at each time point. (TIF) [file pone.0033526.s001.tif]

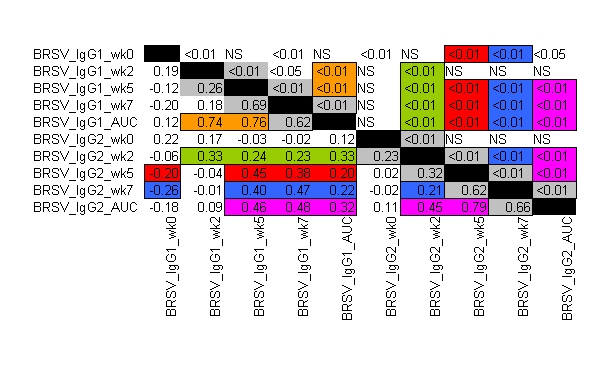

Supplement: Table S2 — Correlations within and between the immune responses to the BRSV vaccine. The x and y axes consist of each week of the BRSV study [27]. Correlations are located below the black shaded boxes. Above the black shaded boxes is the significance of each correlation. The horizontal orange (IgG1 AUC measurement), green (IgG2 levels two post vaccination), red (IgG2 levels 5 weeks post vaccination), blue (IgG2 levels 7 weeks post vaccination) and purple (IgG2 AUC measurement) shaded boxes represent the significant correlations throughout the BRSV study. The vertical coloured shaded boxes highlight the corresponding significance of the correlations. (TIF) [file pone.0033526.s002.tif]

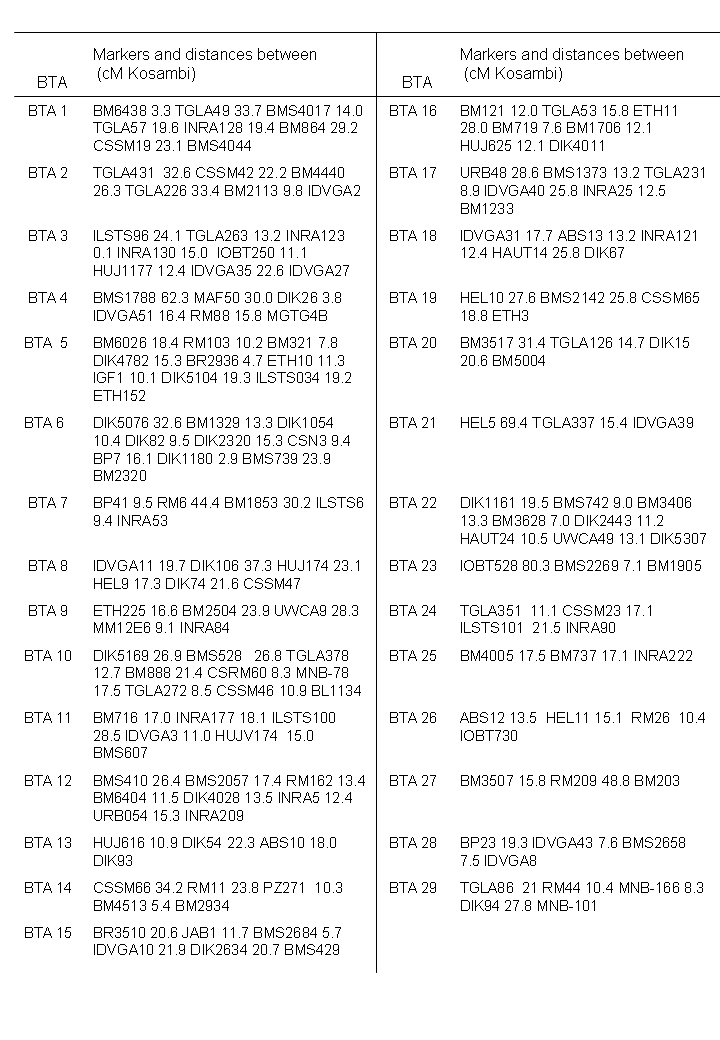

Supplement: Table S3 — Linkage map. Marker distances (cM Kosambi) are shown for the sex-average maps built for the Charolais×Holstein population used in this study. (TIF) [file pone.0033526.s003.tif]
